# A five-plex Hepatic Oncochip reveals EMT triplet correlated with BAP31 in liver cancer

**DOI:** 10.3389/fcell.2024.1478444

**Published:** 2025-01-06

**Authors:** Youjia Kong, Tengfei Zhuang, Xvshen Ding, Sirui Cai, Weijie Ding, Xiaoxiao Zhang, Yubo Sun, Bingquan Zhou, Yuanjie Sun, Shuya Yang, Xiyang Zhang, Kun Yang, Dongbo Jiang

**Affiliations:** ^1^ Department of Immunology, Basic Medicine School, Air-Force Medical University (The Fourth Military Medical University), Xi’an, Shaanxi, China; ^2^ The Key Laboratory of Bio-Hazard Damage and Prevention Medicine, Basic Medicine School, Air-Force Medical University (The Fourth Military Medical University), Xi’an, Shaanxi, China; ^3^ Department of Neurosurgery, Tangdu Hospital, Air-Force Medical University (The Fourth Military Medical University), Xi’an, Shaanxi, China; ^4^ Department of Information, Medical Supplies Center of PLA General Hospital, Beijing, China; ^5^ Military Medical Innovation Center, Air-Force Medical University (Fourth Military Medical University), Xi’an, Shaanxi, China

**Keywords:** liver cancer, multiplex immunohistochemistry, epithelialmesenchymal transition, biomarkers, fluorescence spectroscopy

## Introduction

Primary liver cancer, with 906,000 new cases, ranks sixth in growth among malignant tumors. Additionally, with 830,000 deaths, it ranks third in terms of mortality (Sung et al., 2021). Hepatocellular carcinoma (HCC), which is the most important form of primary liver cancer, accounts for approximately 90% of liver cancer cases (Anwanwan et al., 2020). A variety of risk factors can contribute to the development of primary liver cancer, including hepatitis B virus (HBV) infection, hepatitis C virus (HCV) infection, fibrotic chronic liver damage, aflatoxin B1, and excessive alcohol consumption (Akinyemiju et al., 2017; European association for the study of the liver and European organisation for research and treatment of cancer, 2012). The progression of HCC evolves continuously from dysplastic lesions with minor genetic mutations to the late stages of HCC, displaying significant molecular heterogeneity involving numerous molecules (Marquardt et al., 2015). The extensive tumor heterogeneity across multiple stages of HCC development hinders patient stratification for effective treatment (Giannelli et al., 2016). Therefore, exploring the tumor heterogeneity of HCC would help stratify patients for effective treatment.

The tumor transformation of HCC usually originates from hepatocytes and progenitor cells, both of which are epithelial cell types. The plasticity changes in these epithelial cells commonly known as epithelial-to-mesenchymal transition (EMT) increase the complexity of cellular heterogeneity (Giannelli et al., 2016). The EMT program in cancer cells can be transiently or stably activated to varying extents during invasion and metastasis. High expression of adhesion molecules can increase cell migration ability and invasiveness. A significant body of evidence illustrates that the EMT plays an important role in cancer invasion and metastasis (Nieto et al., 2016; Thiery et al., 2009; Thiery, 2002; Hanahan and Weinberg, 2011). By analyzing various EMT phenotypes in malignant epithelial hepatocytes, researchers could estimate the complexity and cellular heterogeneity of HCC. Few studies have investigated several EMT markers in a large number of biopsies, making it difficult to identify the occurrence of EMT based on a single marker alone (Yang et al., 2009). E-cadherin together with occludin or cytokeratins represents the most commonly used markers for epithelial features, while N-cadherins and vimentin are markers for mesenchymal features (Thiery et al., 2009). Hence, in this study, E-cadherin, N-cadherins, and vimentin (EMT triplet) were selected to characterize the occurrence of EMT.

B-cell receptor-associated protein 31 (BAP31) is named for its association with the B-cell receptor component immunoglobulin D. BAP31 exhibits an apparent molecular weight of 31 kDa, a characteristic that is clearly observed on a denaturing electrophoresis gel (Kim et al., 1994). BAP31 has been identified as a cancer antigen (Dang et al., 2018). It is overexpressed in cancer tissues compared to healthy adjacent tissues, and it holds promise as a prognostic biomarker for several different types of cancer (Dang et al., 2018; Chen et al., 2019; Wang et al., 2020; Xu et al., 2019). In our previous study, using mIHC and multispectral imaging techniques, we demonstrated that BAP31 promotes cell proliferation by interacting with Serpin Family E Member 2 (SERPINE2) in hepatocellular carcinoma (HCC) (Zhang et al., 2020). Only a few studies have reported on the role of BAP31 in activating invasion and metastasis. Recent studies have demonstrated that BAP31 can induce epithelial–mesenchymal transition (EMT) by enhancing the expression of the EMT-related factor Snail and decreasing the content and membrane distribution of E-cadherin (Liu et al., 2021). However, it remains scarce how BAP31 induces the entire EMT process. The current study reported a multi-molecule staining dataset of EMT triplet and BAP31 to estimate tumor heterogeneity and preliminarily explored the relationship between BAP31 and EMT.

Conventional immunohistochemistry techniques have several limitations. Multiplex immunohistochemistry (mIHC) technology allows for the simultaneous detection of multiple markers on a single tissue section, providing a new method for comprehensive studies of cell composition, cell function, and intercellular interactions. Moreover, the use of multiplex immunohistochemistry (mIHC) (Tan et al., 2020). Through the use of mIHC, EMT triplets and BAP31 in liver cancer can be elucidated together.

Here, we present our dataset containing numerous images and analyzing data from a digitally scanned high-resolution tissue microarray (TMA) with 138 samples, termed LV138. The TMA was stained for HE and other specific biomarkers, such as BAP31, E-cadherins, N-cadherins, vimentin, and 4,6-diamidino-2-phenylindole (DAPI). Each sample was accompanied by clinical data, pathologist annotations, and staging information. Cell and tissue segmentation, as well as the expression of specific biomarkers, were performed using inForm Advanced image analysis software (inForm 2.6, Akoya). The results of each step were compiled and formed part of the dataset. The utility of our datasets was confirmed by preliminary statistical analysis. Application of dataset can prevent a multitude of repetitive operations and offers an insightful approach for investigating molecular heterogeneity of HCC.

## Materials and methods

### Study cohort

This patient group consisted of 138 primary liver cancer patients aged between 21 and 98 years, including 37 women and 101 men. We obtained 138 tissue samples from these individuals and stored them in a TMA-LV138. The study involving human participants was reviewed and approved by the Ethics Committee of the Fourth Military Medical University. Our samples were derived from a commercial tissue microarray (TMA), and the supplier ensured that informed consent was obtained from all patients during the data collection process.

The original primary liver cancer tissue microarray (TMA), which was stained with hematoxylin and eosin (HE), was independently evaluated and analyzed by at least two pathologists. This meticulous evaluation led to the identification of 138 viable core samples. Each of these core samples was annotated with essential details such as the diagnosis, grade, TNM classification, stage, and type of cancer. Additionally, each core includes basic patient information and a unique identifier for accurate reference.

To enhance visualization, the LV138 was scanned using an Aperio GT 450 scanner from Leica Biosystems (USA). The high-resolution scanning process produced SVS images that were subsequently saved in TIF format, ensuring clear and detailed representation of the tissue samples.

### Ethics

The studies involving humans were approved by the institutional review board of the Fourth Military Medical University. The studies were conducted in accordance with the local legislation and institutional requirements. The human samples used in this study were acquired from a by-product of routine care or industry.

### Multiplex immunofluorescence staining

**FIGURE 1:**
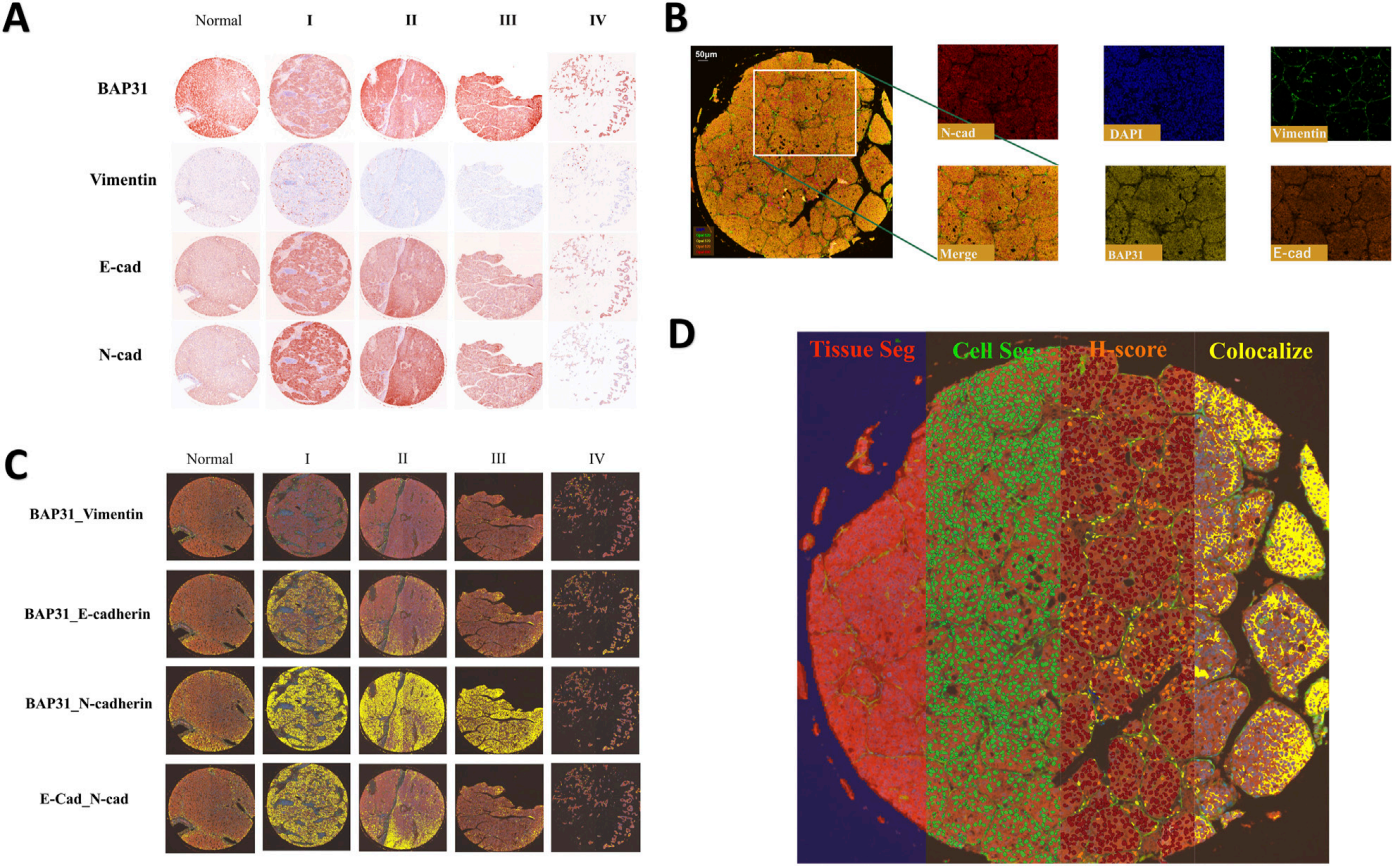
**(A)**: Four biomarkers of normal cores and HCC cores at different stages stained by mIHC technology; a darker color indicates higher biomarker expression. **(B)** The stained core was multispectral split, and the merged image was split into five images: Opal520-stained vimentin (green), Opal570-stained BAP31 (brown), Opal620-stained E-cadherin (orange), Opal690-stained N-cadherin (red) and DAPI (blue). **(C)** Colocalization of E-cadherin and N-cadherin. Colocalization analysis was subsequently performed. The percentage colocalization area (golden) was calculated. **(D)** All three steps to train the inForm for segmentation and the calculation of the H-score. From left to right are the tissue segmentation, cell segmentation and H-score. Each step is further analyzed based on the previous step. Different tissues were first divided into cancer (red), stromal (green), and background (blue) tissues. Then, the cells were segmented. According to the biomarker expression level, we set thresholds to divide cells into 4 grades (blue indicates no expression, yellow indicates +1 expression, orange indicates +2 and brown indicates +3), and the H-score was calculated. The percentage of the colocalization area (golden) was calculated.

Multiplexed tyramide signal amplification (TSA) immunofluorescence was performed on the LV138 TMA to simultaneously visualize multiple biomarkers on a single section using Opal 7-plex technology (Akoya). Tyramide Signal Amplification (TSA) is an enzymatic detection method based on horseradish peroxidase (HRP). The principle involves the covalent binding of fluorescently labeled tyramine to the tyrosine residues of the target protein under the catalysis of HRP, thereby labeling the target protein with specific fluorescence (Faget and Hnasko, 2015). The original slides of the LV138 TMA were processed into 5-μm-thick sections. The slides were deparaffinized in xylene and rehydrated in an ethanol gradient. The mIHC staining includes four sequential cycles, each specifically targeting one of the following molecular markers: Vimentin, BAP31, E-cadherin, and N-cadherin. At the start of each staining cycle, the slides are first immersed in either EDTA buffer (pH 9.0) or citrate buffer (pH 6.0). The reaction container with the slides is then placed in a microwave for heat-induced antigen retrieval and stripping. To minimize non-specific binding, we apply a 5% Bovine Serum Albumin (BSA) blocking solution to the slides and allow it to rest for 15 min. According to a pre-established and optimized protocol, during each round of staining, the slides are first incubated with primary antibodies—anti-Vimentin (Proteintech; 10366-1-AP), anti-BAP31 (FMU-BAP31-2) (Zhang et al., 2020), anti-E-cadherin (Proteintech; 60335-1-IG), anti-N-cadherin (Proteintech; 66219-1-IG)—followed by incubation with HRP-conjugated secondary antibodies (Akoya; Opal Polymer HRP Ms + Rb; ARH1001EA) to detect specific molecular markers. Afterwards, during each round of staining, the slides are incubated with fluorophore-conjugated tyramide—Opal 520, Opal 570, Opal 620, and Opal 690—for 10 min to visualize the corresponding molecular markers. Once a TSA staining round is complete, the same method used previously is applied for heat-induced antibody stripping and antigen retrieval. Finally, the cell nuclei are stained with 4,6-diamidino-2-phenylindole (DAPI; Sigma-Aldrich, St. Louis, Missouri, USA; Catalog No. D9542), marking the completion of the entire staining process. The slides were scanned using Vectra 3.0 (Akoya) to acquire multispectral images. All the samples are shown in the resulting images and an example of these images is shown in Figure 1A.

### Multispectral image analysis

The multispectral images were unmixed by advanced image analysis software (inForm 2.6.0, Akoya). Example unmixed pictures are shown in Figure 1B. In addition to generating deconvoluted images of individual biomarkers, we performed colocalization analysis of the deconvolved pixels and discovered correlations among these biomarkers. Example of colocalization images are shown in Figure 1C. To score biomarker expression levels in cancer cells, we selected representative multispectral images for algorithm training. Subsequently, the inForm software executed image segmentation and scoring utilizing an AI-driven feature recognition algorithm. The scoring process comprises three automated stages: tissue segmentation, cell segmentation, and biomarker expression scoring. During tissue segmentation, after training the inForm software with specific samples from each category, the software automatically segments each image into a cancer region (red), matrix region (green), and background (blue) using batch and merge functions. Once the images were reviewed, merged, and exported, the data were consolidated into a single dataset for analysis, and the percentages of different tissues were determined. For cell segmentation, we established a minimum nuclear size of 25 nm based on the nuclear dye (DAPI) to segment individual cells from the tissue. Although the InForm software is capable to quantify single-cell staining intensity, it cannot identify the cell types such as cancer cells, infiltrated immune cells, and other stromal cells based on 5-plex staining. In the current study, conditions could only allow for an objective conclusion at tissue level. It was the reason that after cell segmentation we selected the histochemical score (H-score) to quantify the staining intensity of four markers. We selected a subset of samples and manually set three thresholds, allowing the inForm software to automatically ascertain the expression levels of various biomarkers in the slides using the same positive threshold settings. Samples were classified into four categories based on biomarker expression levels (blue indicates no expression, yellow indicates +1 expression, orange indicates +2, and brown indicates +3). H-score was calculated using the following formula: H-score = (percentage of high fluorescence intensity) × 3 + (percentage of median fluorescence intensity) × 2 + (percentage of low fluorescence intensity) × 1. By computing the weighted average between ranks and proportions of cells exhibiting various expression levels, we obtained H-scores to quantify the expression of biomarkers. An example of all three steps is shown in Figure 1D.

### Data records

**FIGURE 2:**
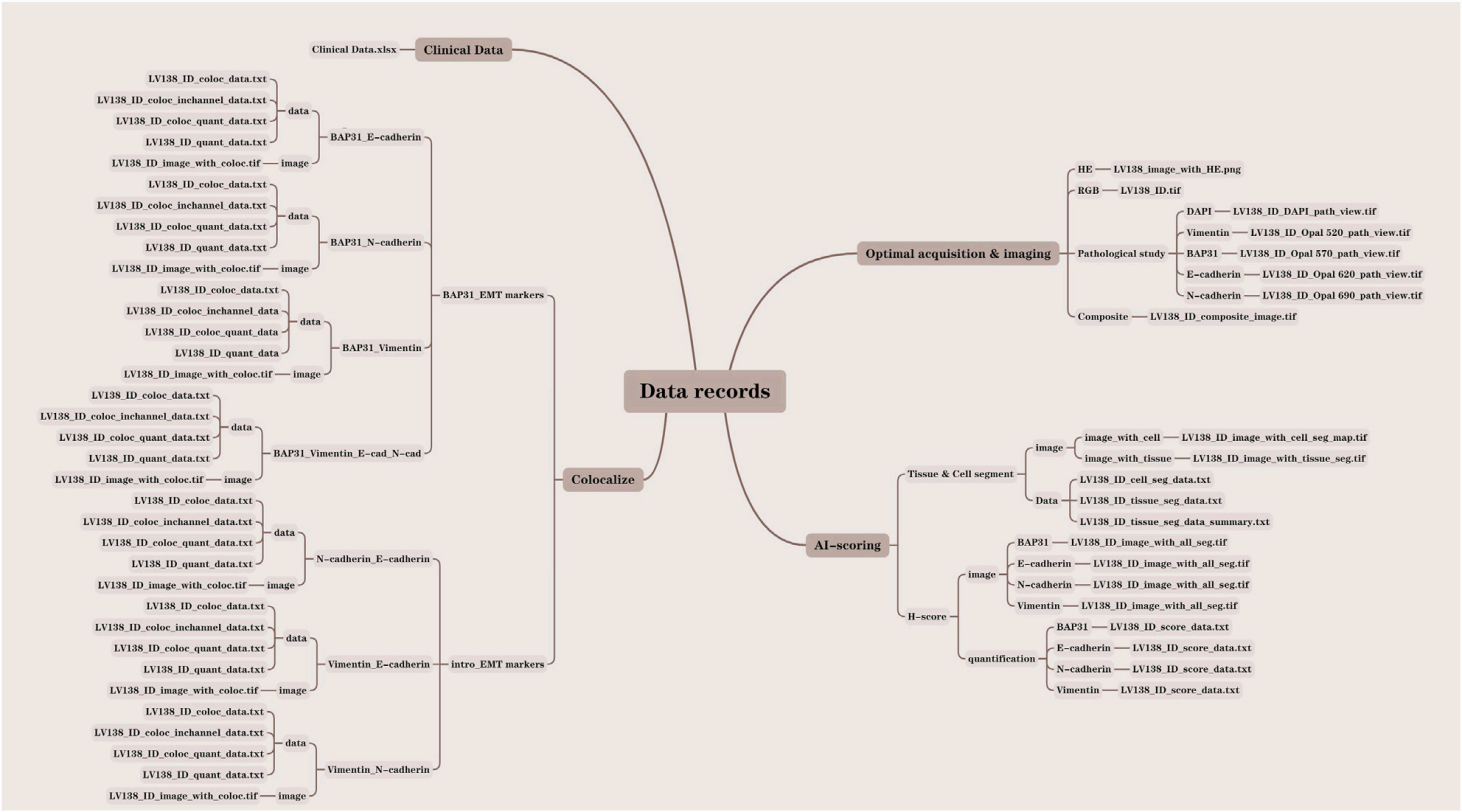
Detailed structure drawing of the data record.

In this study, we analyzed a total of 138 core samples, comprising 118 hepatocellular carcinoma (HCC) cores, 10 cholangiocarcinoma cores, and 10 normal liver cores. All pertinent data were meticulously documented within the LV138 dataset. The data records were systematically organized into four distinct folders: clinical data, optical acquisition and imaging, Artificial Intelligence (AI) scoring, and colocalization. Each file was methodically named according to the LV138 system, incorporating the file ID and data type to facilitate comprehension of our data structure. To further elucidate our organizational approach, we developed a data result diagram showed in Figure 2 and appended a file type suffix to each file name (e.g., LV138_ID_coloc_data.txt).

### Clinical data

The clinical data of the patients are stored in Clinical Data. xlsx, including pathologist annotations, age, sex, diagnosis, grade and unique identifiers for each sample.

### Optical acquisition and imaging

The optical acquisition and imaging process began with scanning the glass slides using the Vectra 3.0 (Akoya) system to obtain raw images in the IM3 format. The raw images were then analyzed using the inForm 2.6.0 software to derive and export RGB images, pathology study images, and composite material images. The optical acquisition and imaging folder consists of four sections: HE, RGB, pathology study, and composite material. The LV138with_HE.png file, stored in the HE folder, contains the original pathological section images of HE staining for all 138 samples. The LV138_ID.tif file is stored in the RGB folder. The LV138_ID_composite_image.tif file, stored in the composite folder, contains 138 composite images used to analyze the expression levels of four markers (Vimentin, BAP31, E-Cadherin, N-Cadherin) across all 138 cores. The pathology study section includes five sub-folders for Vimentin, BAP31, E-Cadherin, N-Cadherin, and DAPI, containing files named LV138_ID_DAPI_path_view.tif, LV138_ID_Opal_520_path_view.tif, LV138_ID_Opal_570_path_view.tif, LV138_ID_Opal_620_path_view.tif, and LV138_ID_Opal_690_path_view.tif, respectively.

### AI scoring

The images were further analyzed using machine learning for additional interpretation. The inForm 2.6.0 software performed tissue and cell segmentation as well as histochemical scoring of markers, producing outputs of both images and analysis data. The AI scoring folder comprises two sections: Tissue & Cell Segment and H-score. The Tissue & Cell Segment section includes two sub-folders: image and data. The files LV138_IDwith_cell_seg_map.tif and LV138_IDwith_tissue_seg_map.tif are stored in the image sub-folder of Tissue & Cell Segment. The files LV138_ID_cell_seg_data.txt, LV138_ID_tissue_seg_data.txt, and LV138_ID_tissue_seg_data_summary.txt are stored in the data sub-folder of Tissue & Cell Segment. The H-score section includes two sub-folders: image and quantification. The image sub-folder contains Vimentin, BAP31, E-Cadherin, N-Cadherin, and the file is stored as LV138_IDwith_all_seg.tif. The quantification sub-folder contains Vimentin, BAP31, E-Cadherin, N-Cadherin, and the file is stored as LV138_ID_score_data.txt.

### Colocalization

To further understand the influence of joint function of biomarks, the colocalizations of biomarkers were analyzed, and the results were divided into two categories, BAP31-EMT markers and EMT markers. The BAP31-EMT marker category includes the colocalization analysis results of each EMT marker with BAP31. The results were divided into four groups: BAP31_E-cadherin, BAP31_N-cadherin, BAP31_Vimentin, and BAP31_E-cad_N-cad_Vimentin. The files, including coloc_data.txt, coloc_quant_data.txt, and quant_data.txt, were stored in the data subfolders, and coloc. tif was stored in the image subfolders.

The EMT markers category includes the colocalization analysis results between each pair of EMT markers. The results were divided into the following groups: N-cadherin_E-cadherin, Vimentin_E-cadherin, and Vimentin_N-cadherin. The files, including coloc _data.txt, coloc_quant_data.txt, and quant_data.txt, were stored in the data subfolders, and coloc. tif was stored in the image subfolders.

### Validation and application

For the preliminary analysis of the data, the datasets were analyzed using GraphPad Prism 10 software (San Diego, CA, United States). The Pearson correlation test was conducted to explore the correlations between four biomarkers, during which the correlation coefficients and P values were calculated, and scatter diagrams were generated. Analysis of variance (ANOVA) was performed to test for the presence of significant differences in different grades, and the H-score was used to represent expression levels. For non-normally distributed rank/ordered variables and data, the Kruskal‒Wallis test was used. All P values were two-sided, and a P values less than 0.05 were considered to indicate statistical significance.

## Results

ANOVA was used to compare the expression levels of BAP31 across different grades of cancer and in normal liver tissue. Notably, BAP31 expression was significantly greater in cancerous tissue than in normal liver tissue, indicating a strong correlation between elevated BAP31 expression and poor prognosis. Pearson correlation analysis revealed a significant relationship between the expression of N-cadherin and E-cadherin in liver cancer and normal liver tissues, with a Pearson’s r value of 0.7749 and a P-value less than 0.0001. This finding aligns with the results from GEPIA2 (Figure GEPIA 2 - Copyright ^©^ 2018) (http://gepia2.cancer-pku.ac.cn), where the Pearson’s r value was 0.4 with a P-value of 0. To further investigate the correlation between N-cadherin and E-cadherin expression in various tissues, we examined their relationship in normal liver and cancer tissues using the same methodology. The results indicated a significant correlation between a normal liver (Pearson’s r = 0.7204, *p* = 0.0188) and liver cancer (Pearson’s r = 0.7723, *p* < 0.0001). However, no significant correlation was found between the two biomarkers in cholangiocarcinoma (*p* = 0.7475). These findings were corroborated by data from GEPIA2 (http://gepia2.cancer-pku.ac.cn).

Contrary to the traditional view of decreased E-cadherin and increased N-cadherin, the strong correlation between N-cadherin and E-cadherin suggests that the conventional epithelial–mesenchymal transition (EMT) model may not be applicable for characterizing hepatocellular carcinoma, but its applicability to cholangiocarcinoma remains to be explored. Recent multicenter study have shown that the expression of N- and E-cadherin are markers for normal hepatocytes and cholangiocytes, respectively, and that the expression of E- and N-cadherin is retained in HCC and intrahepatic cholangiocarcinoma (iCCA) (Gerber et al., 2024; Straub et al., 2011). His research aligns with our findings, further validating the credibility and value of our dataset.

Additionally, the colocalization of E-cadherin and BAP31 was analyzed using ANOVA across four grades of cancer, yielding a p-value of 0.0104. The colocalization analysis showed the extent of the overlapping area between the two biomarkers within the entire tissue core, suggesting a potential relationship between the degree of interaction between the biomarkers and the progression of cancer. Notably, survival data from the TCGA database (https://www.cancer.gov/aboutnci/organization/ccg/research/structural-genomics/tcga) indicate a trend toward diminished overall survival with increased transcription of the corresponding mRNA. This observation supports the validity of our database for survival assessments.

Our findings suggest that multispectral analysis can significantly contribute to the diagnosis of liver cancer. Further studies based on a larger patient cohort are warranted. Moreover, incorporating survival outcomes could provide a critical indicator for predicting survival rates.

## Data Availability

The datasets presented in this study can be found in online repositories. The names of the repository/repositories and accession number(s) can be found in the article/Supplementary Material.

## References

[B1] Akinyemiju T. Abera S. Ahmed M. Alam N. Alemayohu M. A. Allen C. (2017). The burden of primary liver cancer and underlying etiologies from 1990 to 2015 at the global, regional, and national level: results from the global burden of disease study 2015. Jama Oncol. 3, 1683–1691. 10.1001/jamaoncol.2017.3055 28983565 PMC5824275

[B2] Anwanwan D. Singh S. K. Singh S. Saikam V. Singh R. (2020). Challenges in liver cancer and possible treatment approaches. Biochim. Biophys. Acta Rev. Cancer 1873, 188314. 10.1016/j.bbcan.2019.188314 31682895 PMC6981221

[B3] Chen J. Guo H. Jiang H. Namusamba M. Wang C. Lan T. (2019). A BAP31 intrabody induces gastric cancer cell death by inhibiting p27(kip1) proteasome degradation. Int. J. Cancer 144, 2051–2062. 10.1002/ijc.31930 30338855

[B4] Dang E. Yang S. Song C. Jiang D. Li Z. Fan W. (2018). BAP31, a newly defined cancer/testis antigen, regulates proliferation, migration, and invasion to promote cervical cancer progression. Cell Death Dis. 9, 791. 10.1038/s41419-018-0824-2 30022068 PMC6052025

[B5] European association for the study of the liver, and European organisation for research and treatment of cancer (2012). EASL-EORTC clinical practice guidelines: management of hepatocellular carcinoma. J. Hepatol. 56, 908–943. 10.1016/j.jhep.2011.12.001 22424438

[B6] Faget L. Hnasko T. S. (2015). Tyramide signal amplification for immunofluorescent enhancement. Methods Mol. Biol. 1318, 161–172. 10.1007/978-1-4939-2742-5_16 26160574

[B7] Gerber T. S. Ridder D. A. Goeppert B. Brobeil A. Stenzel P. Zimmer S. (2024). N-cadherin: a diagnostic marker to help discriminate primary liver carcinomas from extrahepatic carcinomas. Int. J. Cancer 154, 1857–1868. 10.1002/ijc.34836 38212892

[B8] Giannelli G. Koudelkova P. Dituri F. Mikulits W. (2016). Role of epithelial to mesenchymal transition in hepatocellular carcinoma. J. Hepatol. 65, 798–808. 10.1016/j.jhep.2016.05.007 27212245

[B9] Hanahan D. Weinberg R. A. (2011). Hallmarks of cancer: the next generation. Cell 144, 646–674. 10.1016/j.cell.2011.02.013 21376230

[B10] Kim K. M. Adachi T. Nielsen P. J. Terashima M. Lamers M. C. Kohler G. (1994). Two new proteins preferentially associated with membrane immunoglobulin D. Embo J. 13, 3793–3800. 10.1002/j.1460-2075.1994.tb06690.x 8070407 PMC395292

[B11] Liu T. Yu J. Ge C. Zhao F. Miao C. Jin W. (2021). B-cell receptor-associated protein 31 promotes metastasis *via* AKT/β-Catenin/Snail pathway in hepatocellular carcinoma. Front. Mol. Biosci. 8, 656151. 10.3389/fmolb.2021.656151 34179078 PMC8231437

[B12] Marquardt J. U. Andersen J. B. Thorgeirsson S. S. (2015). Functional and genetic deconstruction of the cellular origin in liver cancer. Nat. Rev. Cancer 15, 653–667. 10.1038/nrc4017 26493646

[B13] Nieto M. A. Huang R. Y. Jackson R. A. Thiery J. P. (2016). EMT: 2016. Cell 166, 21–45. 10.1016/j.cell.2016.06.028 27368099

[B14] Straub B. K. Rickelt S. Zimbelmann R. Grund C. Kuhn C. Iken M. (2011). E-N-cadherin heterodimers define novel adherens junctions connecting endoderm-derived cells. J. Cell Biol. 195, 873–887. 10.1083/jcb.201106023 22105347 PMC3257573

[B15] Sung H. Ferlay J. Siegel R. L. Laversanne M. Soerjomataram I. Jemal A. (2021). Global cancer statistics 2020: GLOBOCAN estimates of incidence and mortality worldwide for 36 cancers in 185 countries. Ca Cancer J. Clin. 71, 209–249. 10.3322/caac.21660 33538338

[B16] Tan W. Nerurkar S. N. Cai H. Y. Ng H. Wu D. Wee Y. (2020). Overview of multiplex immunohistochemistry/immunofluorescence techniques in the era of cancer immunotherapy. Cancer Commun. (Lond) 40, 135–153. 10.1002/cac2.12023 32301585 PMC7170662

[B17] Thiery J. P. (2002). Epithelial-mesenchymal transitions in tumour progression. Nat. Rev. Cancer 2, 442–454. 10.1038/nrc822 12189386

[B18] Thiery J. P. Acloque H. Huang R. Y. Nieto M. A. (2009). Epithelial-mesenchymal transitions in development and disease. Cell 139, 871–890. 10.1016/j.cell.2009.11.007 19945376

[B19] Wang J. Jiang D. Li Z. Yang S. Zhou J. Zhang G. (2020). BCAP31, a cancer/testis antigen-like protein, can act as a probe for non-small-cell lung cancer metastasis. Sci. Rep. 10, 4025. 10.1038/s41598-020-60905-7 32132574 PMC7055246

[B20] Xu K. Han B. Bai Y. Ma X. Y. Ji Z. N. Xiong Y. (2019). MiR-451a suppressing BAP31 can inhibit proliferation and increase apoptosis through inducing ER stress in colorectal cancer. Cell Death Dis. 10, 152. 10.1038/s41419-019-1403-x 30770794 PMC6377610

[B21] Yang M. H. Chen C. L. Chau G. Y. Chiou S. H. Su C. W. Chou T. Y. (2009). Comprehensive analysis of the independent effect of twist and snail in promoting metastasis of hepatocellular carcinoma. Hepatology 50, 1464–1474. 10.1002/hep.23221 19821482

[B22] Zhang X. Jiang D. Yang S. Sun Y. Liu Y. Shi J. (2020). BAP31 promotes tumor cell proliferation by stabilizing SERPINE2 in hepatocellular carcinoma. Front. Cell Dev. Biol. 8, 607906. 10.3389/fcell.2020.607906 33363167 PMC7759511

