# Supplementary material for: A five-plex Hepatic Oncochip reveals EMT triplet correlated with BAP31 in liver cancer
Source: Front Cell Dev Biol. 2025 Jan 6;12:1478444. doi: 10.3389/fcell.2024.1478444 (PMC11743502; doi:10.3389/fcell.2024.1478444)
Supplement: Supplementary file 2 [file DataSheet1.docx]

Supplementary Material

**Supplementary Figure 1.**The results are shown in graphs and compared with data from GEPIA2. **(A)** BAP31 expression and colocalization of BAP31 and E-cadherin are obviously different in the four stages. **(B)**Scatter diagram acquired from our dataset. In the three groups, Pearson correlation analysis between E-cadherin and N-cadherin all show significant statistical differences. **(C)** Scatter diagram acquired from GEPIA2. In the three groups, Pearson correlation analysis between E-cadherin and N-cadherin all show significant statistical differences. **(D)** The statistical results of overall survival under the influence of one signature (BAP31, CDH1 and CDH2) in the joint cohort of LIHC from TCGA.


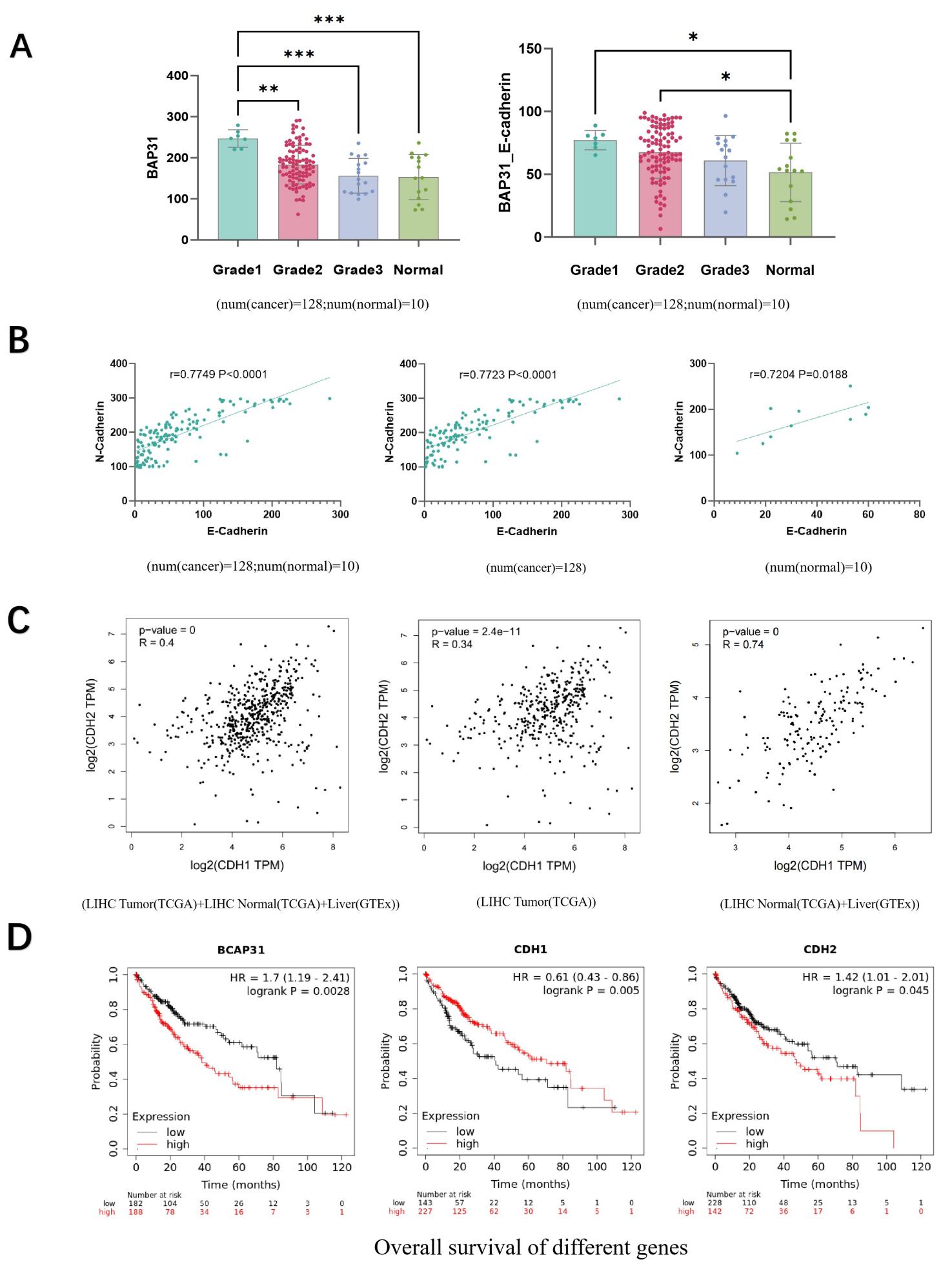


**Supplementary Datasets.**The datasets presented in this study can be found in online repositories. It could be freely accessed at: <https://www.jianguoyun.com/p/DU82cb4Q6PSfDBjYm9EFIAA.> If the link is broken or inaccessible, please contact the author at 2899580571@qq.com.
